# Multidimensional Evaluation of the Efficacy of a Comprehensive Ayurveda Approach as an Add-On to Conventional Therapy in Children With Autism Spectrum Disorder: Protocol for a Randomized, Open-Label, Parallel-Group, Active-Controlled Clinical Trial

**DOI:** 10.2196/99910

**Published:** 2026-10-09

**Authors:** Vidyashree Anchan, Thejaswini C, Srinibash Sahoo, Sonam Chandhok, B C S Rao, Renu Singh, Meghna P P, Sindhu C V, Rakshitha Kotian, N Srikanth

**Affiliations:** 1 Central Council for Research in Ayurvedic Science, 61-65, opp. D’ Block, D Block, Janakpuri Institutional Area, Janakpuri, New Delhi, Delhi, 110058, India, 91 8971859797

**Keywords:** autism spectrum disorder, ASD, Ayurveda, Saraswata Ghrita, Ayush Manas, randomized controlled trial, RCT, study protocol

## Abstract

**Background:**

Autism spectrum disorder (ASD) is a heterogeneous neurodevelopmental disorder characterized by impairments in social communication, restricted and repetitive behaviors, and deficits across multiple developmental domains. Although conventional interventions, including speech, behavioral, and occupational therapies, improve functional outcomes, many children continue to experience persistent cognitive and adaptive impairments. Ayurvedic formulations such as *Saraswata Ghrita* and *Ayush Manas* possess neuroprotective, anti-inflammatory, antioxidant, and cognition-enhancing properties, but robust clinical evidence supporting their adjunctive use in ASD is limited.

**Objective:**

The study aims to evaluate the efficacy of a comprehensive Ayurveda intervention as an adjunct to conventional therapy in children with ASD by assessing changes in behavioral symptoms, intellectual functioning, adaptive behavior, parental quality of life, and exploratory inflammatory biomarkers.

**Methods:**

This randomized, open-label, parallel-group, active-controlled clinical trial will enroll 80 children aged 6 to 15 years diagnosed with ASD according to *DSM-5* (*Diagnostic and Statistical Manual of Mental Disorders, Fifth Edition*) criteria at the Central Ayurveda Research Institute (CARI), Bengaluru, in collaboration with selected autism therapy centers. Participants will be randomized in a 1:1 ratio to receive either conventional therapy alone or conventional therapy combined with *Saraswata Ghrita* and *Ayush Manas* for 6 months. Outcome assessments will be performed at predefined intervals using the Autism Treatment Evaluation Checklist (ATEC), Indian Scale for Assessment of Autism (ISAA), Wechsler Intelligence Scale for Children-Fourth Edition (WISC-IV), Vineland Adaptive Behavior Scales, Third Edition (VABS-III), and Quality of Life in Autism (QoLA) questionnaire. Exploratory laboratory assessments include high-sensitivity C-reactive protein, interleukin-6, and tumor necrosis factor-alpha (TNF-α). Safety will be evaluated through adverse event monitoring and laboratory investigations. Statistical analysis will be performed after data verification. Data normality will be assessed using the Shapiro-Wilk test. Variables will be represented as frequencies (%), mean (SD), or median with IQR. Preintervention and postintervention comparisons will use repeated-measures ANOVA or the Friedman test, as appropriate, with significance set at *P*<.05, using STATA software (version 18.0; StataCorp LLC).

**Results:**

Ethical approval was obtained before study initiation, and the trial was registered with the Clinical Trials Registry–India (CTRI/2025/04/085035). Participant recruitment commenced on December 3, 2025. As of July 17, 2026, 20 participants had been enrolled and completed baseline assessments; 14 had completed the 90-day follow-up, and 2 had completed the 6-month intervention. Recruitment is expected to continue until August 2027, followed by completion of participant follow-up, data analysis, and dissemination of study findings.

**Conclusions:**

The study will provide high-quality evidence regarding the effectiveness of integrating Ayurveda with conventional therapy for children with ASD. By simultaneously evaluating behavioral, cognitive, adaptive, quality-of-life, and exploratory biological outcomes, the trial is expected to strengthen the evidence base for multidisciplinary integrative care and inform the development of standardized treatment strategies for ASD.

## Introduction

### Terminological Construct of ASD

Autism spectrum disorder (ASD) is classified by the World Health Organization in *ICD-11* (*International Classification of Diseases, 11th Revision*; code: 6A02) under neurodevelopmental disorders and is defined by persistent deficits in initiating and sustaining reciprocal social interaction and social communication and by a range of restricted, repetitive, and inflexible patterns of behavior, interests, or activities [1]. The *DSM-5* (*Diagnostic and Statistical Manual of Mental Disorders, Fifth Edition*), as published by the American Psychiatric Association, similarly conceptualizes ASD, with the diagnostic criteria largely consistent with *ICD-11* [2]. ASD presents with wide variability in symptom severity and functional impairment, necessitating individualized therapeutic approaches.

### Clinical Landscape and Epidemiological Trajectory of ASD

Recent epidemiological data indicate a continuing rise in ASD prevalence worldwide. According to the Centers for Disease Control and Prevention, approximately 1 in 31 children in the United States has been diagnosed with ASD [3]. Similar trends have been reported globally, including in Asian countries. In India, increasing awareness, improved diagnostic services, and expanded epidemiological surveillance have contributed to greater recognition of ASD. However, despite this increasing disease burden, access to comprehensive, evidence-based interventions remains limited, and universally accepted treatment guidelines addressing the multidimensional nature of ASD are lacking.

### Etiological Complexity and Biochemical Underpinnings

Although the precise etiology of ASD remains incompletely understood, current evidence suggests that its pathogenesis is multifactorial, involving complex interactions among genetic susceptibility, environmental influences, immune dysregulation, oxidative stress, neuroinflammation, and abnormalities of the gut-brain axis [4-7]. Increasing evidence indicates that persistent neuroinflammation and altered immune responses contribute to behavioral and cognitive dysfunction in ASD. Elevated circulating inflammatory mediators, including high-sensitivity C-reactive protein (hs-CRP), interleukin-6 (IL-6), and tumor necrosis factor-alpha (TNF-α), have been consistently reported in individuals with ASD, supporting the hypothesis that inflammatory pathways may influence disease severity and therapeutic response [7]. Although these biomarkers are not diagnostic of ASD, they provide valuable insight into underlying biological mechanisms and may serve as exploratory indicators of treatment response.

Current management of ASD primarily relies on multidisciplinary nonpharmacological interventions, including behavioral, speech-language, occupational, and educational therapies. These interventions improve functional outcomes but often require prolonged implementation and variable caregiver engagement. Pharmacological therapies are generally reserved for associated symptoms such as irritability, aggression, hyperactivity, or sleep disturbances and have limited efficacy in addressing the core deficits of ASD. Furthermore, emerging pharmacological agents, including intranasal oxytocin and N-acetylcysteine, remain investigational, with inconsistent evidence supporting routine clinical use [8]. Consequently, there is growing interest in adjunctive interventions capable of complementing conventional therapy through multiple biological and behavioral mechanisms.

### Ayurvedic Neurotherapeutics and Neuroprotective Synergy

Ayurveda provides a holistic and individualized approach to health that emphasizes restoration of physiological balance through multimodal therapeutic strategies. Although ASD is not explicitly described in classical Ayurvedic literature, many of its clinical manifestations resemble those described under *Unmada*, a condition characterized by disturbances in cognition, behavior, speech, and emotional regulation [9]. Among the Ayurvedic interventions traditionally indicated for cognitive disorders, *Saraswata Ghrita* and the polyherbal formulation *Ayush Manas* have received increasing attention because of their reported neuroprotective, antioxidant, anti-inflammatory, and cognition-enhancing properties. Their constituent herbs, including *Brahmi* (*Bacopa monnieri*), *Ashwagandha* (*Withania somnifera*), *Jyotishmati* (*Celastrus paniculatus*), and *Mandukaparni* (*Centella asiatica*), have demonstrated beneficial effects on neuronal function, oxidative stress, neuroinflammation, and synaptic plasticity in experimental and early clinical studies [10-14]. Therefore, inflammatory biomarkers, including hs-CRP, IL-6, and TNF-α, have been incorporated as exploratory mechanistic outcome measures to evaluate whether adjunctive Ayurvedic therapy is associated with modulation of inflammatory processes alongside improvements in behavioral and functional outcomes. Collectively, these findings provide a biologically plausible rationale for evaluating these formulations as adjuncts to conventional ASD therapy [15,16].

### Multidimensional Outcome Assessment in ASD

As ASD affects multiple domains of functioning, assessment of treatment response requires validated multidimensional outcome measures. Accordingly, this study employs the Autism Treatment Evaluation Checklist (ATEC) as the primary outcome measure and incorporates secondary outcome measures, including the Indian Scale for Assessment of Autism (ISAA) to measure severity across core behavioral domains, the Wechsler Intelligence Scale for Children (WISC) for intellectual ability, the Vineland Adaptive Behavior Scale (VABS), particularly the Daily Living Skills, to determine functional independence, and Quality of Life in Autism Questionnaire (QoLA) to evaluate parental quality of life, and exploratory inflammatory biomarkers. Together, these measures enable comprehensive evaluation of both clinical and potential biological effects of the intervention [17-20].

### Translational Gaps and Advancing Evidence Frontiers

Despite encouraging preclinical evidence and limited clinical observations supporting Ayurvedic interventions in neurodevelopmental disorders, robust randomized controlled trials (RCTs) evaluating their adjunctive role in ASD remain scarce. High-quality evidence is therefore required to determine whether integration of Ayurveda with conventional therapy can improve behavioral, cognitive, adaptive, and family-centered outcomes while also providing insight into potential biological mechanisms.

### Scientific Rationale and Investigational Framework

The study describes the protocol for a randomized, open-label, active-controlled clinical trial evaluating a comprehensive Ayurveda intervention comprising *Saraswata Ghrita* and *Ayush Manas* as an adjunct to conventional therapy in children with ASD. The trial aims to determine whether this integrative approach improves behavioral symptoms, intellectual functioning, adaptive behavior, and parental quality of life while exploring changes in inflammatory biomarkers, including hs-CRP, IL-6, and TNF-α. The findings are expected to strengthen the evidence base for integrative ASD management and inform the development of standardized multidisciplinary treatment strategies.

### Objectives

The primary objective of this study is to evaluate the efficacy of the comprehensive Ayurveda approach as an add-on to the conventional therapy in children with ASD. Additionally, the study aims to assess the intellectual ability and daily living adaptive functioning of the children, as well as the quality of life of their parents.

## Methods

### Trial Design

This study is a randomized, open-label, parallel-group, active-controlled clinical trial with a 1:1 allocation ratio, designed in accordance with the SPIRIT (Standard Protocol Items: Recommendations for Interventional Trials) 2025 guidelines (Checklist 1). The trial evaluates the efficacy of a comprehensive Ayurveda intervention comprising *Saraswata Ghrita* and *Ayush Manas* as an adjunct to conventional therapy in children with ASD. Participants will be allocated to either conventional therapy alone (control group) or conventional therapy combined with the Ayurvedic intervention (intervention group) for 6 months. The schedule of enrollment, interventions, and outcome assessments is presented in Table 1.

**Table 1.** Schedule of study procedures: enrollment, intervention, and assessment.

Activity/assessment	−T1, screening day	T0, baseline and allocation (day 0)	T1, day 30	T2, day 60	T3, day 90	T4, day 120	T5, day 150	T6, day 180 (end of study)
Enrollment								
Eligibility screening	✓							
Informed consent	✓							
Baseline demographics and medical history		✓						
Baseline assessments		✓						
Randomization (1:1, N=80 [40/arm])		✓						
Intervention								
Group 1: Conventional therapy		✓ (start)	✓	✓	✓	✓	✓	✓ (complete)
Group 2: Conventional therapy+Ayurveda		✓ (start)	✓	✓	✓	✓	✓	✓ (complete)
Assessment								
Primary outcome: ATEC^a^	✓							✓
Secondary outcomes: ISAA^b^, WISC^c^, VABS^d^ (Daily Living Skill subscale), and QoLA^e^-parent version	✓ (ISAA)	✓						✓
Safety and adverse events			✓	✓	✓	✓	✓	✓
Concomitant medications			✓	✓	✓	✓	✓	✓
Adherence and compliance			✓	✓	✓	✓	✓	✓
Lab tests and biomarkers	✓	✓						✓
Withdrawal and attrition			✓	✓	✓	✓	✓	✓

a ATEC: Autism Treatment Evaluation Checklist.

b ISAA: Indian Scale for Assessment of Autism.

c WISC: Wechsler Intelligence Scale for Children.

d VABS: Vineland Adaptive Behavior Scales.

e QoLA: Quality of Life in Autism.

### Trial Setting

The trial is being conducted at the Central Ayurveda Research Institute (CARI), Bengaluru, India, in collaboration with selected autism therapy centers that provide standard multidisciplinary ASD care. Conventional interventions, including behavioral, speech-language, occupational, and special education therapies, are delivered at the collaborating centers, whereas study enrollment, clinical assessments, laboratory investigations, dispensing of study medications, and follow-up evaluations are undertaken at CARI.

The study is conducted by a multidisciplinary research team comprising the principal investigator, coinvestigators, Ayurveda physicians, psychologists, pediatricians, laboratory personnel, and trained study coordinators. The principal investigator is responsible for overall trial conduct, protocol compliance, participant safety, and data integrity. Coinvestigators oversee clinical assessments and outcome evaluations, while trained study personnel are responsible for participant recruitment, data collection, documentation, follow-up, and maintenance of study records in accordance with Good Clinical Practice (GCP) and institutional standard operating procedures.

### Participants

Children diagnosed with ASD and receiving conventional therapy at participating autism therapy centers are screened for eligibility before enrollment. Recruitment commenced in December 2025 and will continue until the required sample size is achieved. Written informed consent is obtained from parents or legal guardians before any study-related procedures are performed.

The eligibility criteria were developed to recruit a clinically homogeneous population while ensuring participant safety and minimizing potential confounding factors that could influence behavioral, cognitive, adaptive, or inflammatory outcomes (Textbox 1). The selected age range (6‐15 y) corresponds to the developmental period during which the standardized assessment instruments used in this study are valid and behavioral interventions can be implemented consistently.

Textbox 1. **Eligibility criteria.**

**Inclusion criteria**

- Children aged 6 to 15 years, irrespective of sex.
- Diagnosis of autism spectrum disorder (ASD) according to the *Diagnostic and Statistical Manual of Mental Disorders, Fifth Edition* (*DSM-5*) criteria.
- Currently receiving conventional multidisciplinary therapy at a participating autism therapy center.
- Indian Scale for Assessment of Autism (ISAA) score between 70 and 153, corresponding to mild or moderate ASD severity.
- Not receiving pharmacological treatment specifically for ASD at enrollment.
- Parent or legal guardian willing to provide written informed consent and comply with all study procedures, treatment schedules, and follow-up visits.

**Exclusion criteria**

- Severe physical disability or organic neurological disorders that may interfere with study participation or outcome assessment.
- Significant sensory or motor impairment, including blindness, severe hearing impairment, or cerebral palsy.
- Severe cardiovascular, pulmonary, endocrine, hepatic, renal, or other systemic diseases.
- Congenital brain abnormalities, hydrocephalus, epilepsy, brain or spinal cord tumors, or other major neurological disorders.
- Psychiatric disorders other than ASD, including schizophrenia, bipolar disorder, major depressive disorder, anxiety disorders, or psychotic illnesses.
- Use of medications that may influence behavioral, cognitive, inflammatory, or immunological outcomes, including antipsychotics, antidepressants, psychostimulants, anticonvulsants, benzodiazepines, mood stabilizers, immunosuppressive agents, or other centrally acting medications within 8 weeks before enrollment.
- Participation in another interventional clinical trial within the previous 2 months.

Short-term medications prescribed for acute illnesses that are not expected to influence study outcomes will not constitute grounds for exclusion and will be documented as concomitant medications.

The selected eligibility criteria are intended to minimize clinical heterogeneity while ensuring that changes observed during follow-up can be more reliably attributed to the study intervention rather than to major comorbid conditions or concurrent pharmacological therapies.

### Intervention and Comparator

#### Description of Comparator

Participants allocated to the control group will continue to receive standard multidisciplinary conventional therapy throughout the 6-month study period. Conventional therapy is delivered according to each child’s Individualized Education Program (IEP) by qualified health care professionals, including psychologists, speech-language pathologists, occupational therapists, special educators, and pediatricians.

The conventional intervention comprises behavioral therapy to improve social communication, behavioral regulation, and adaptive functioning; speech and language therapy to enhance receptive, expressive, and pragmatic communication skills; occupational therapy to improve sensory integration, fine motor skills, and activities of daily living; and individualized educational support provided through special education programs.

Because the frequency and intensity of conventional therapies may vary among participants, the type, frequency, and duration of each intervention will be documented at baseline and reviewed at every follow-up visit. Participants will be encouraged to maintain their prescribed conventional therapy program throughout the study to minimize performance bias and facilitate interpretation of treatment outcomes [21,22].

#### Description of Intervention

Participants in the intervention group receive the same conventional therapy, along with *Saraswata Ghrita* (medicated ghee) and *Ayush Manas* tablets, for 6 months. *Saraswata Ghrita* will be administered orally at a dose of 6 g once daily before meals with lukewarm water. *Ayush Manas* tablets, a formulation developed by the Central Council for Research in Ayurvedic Sciences (CCRAS), contain *Brahmi* (*Bacopa monnieri*), *Ashwagandha* (*Withania somnifera*), *Jyotishmati* (*Celastrus paniculatus*), and *Mandukaparni* (*Centella asiatica*) and will be administered orally at a dosage of 2 tablets of 250 mg twice daily after meals with lukewarm water. Both study drugs are Good Manufacturing Practices (GMP)–certified, prepared according to the Ayurvedic Formulary of India, and stored securely under investigator supervision, with detailed records maintained for dispensing and accountability. The Ayurvedic intervention, *Saraswata Ghrita*, was selected based on its classical indications in the Ayurvedic Pharmacopoeia of India (API; part II, volume I, page 85), while *Ayush Manas* is a standardized formulation developed by the CCRAS for conditions involving cognitive and neurobehavioral impairments. The dose and frequency of *Saraswata Ghrita* and *Ayush Manas* were determined based on the recommendations of the API, classical Ayurvedic texts, available preclinical and clinical evidence, and CCRAS and other expert consensus for the pediatric population to ensure safety, therapeutic efficacy, acceptability, treatment adherence, and therapeutic appropriateness in children with ASD.

*Saraswata Ghrita* is traditionally indicated as a *Medhya Rasayana* for promoting cognitive functions, memory, speech, and neurodevelopment, while *Ayush Manas* tablets are indicated for supporting mental health and behavioral regulation. The selected twice-daily dosing regimen of *Ayush Manas* was considered appropriate to provide sustained therapeutic exposure, facilitate caregiver-assisted administration, and optimize treatment adherence throughout the study period. The duration of the intervention was chosen to allow adequate time for the formulations to exert their proposed neuroprotective, antioxidant, anti-inflammatory, and immunomodulatory effects and to permit meaningful assessment of changes in behavioral, cognitive, adaptive, and quality-of-life outcomes.

### Fidelity and Participant Adherence

To ensure consistent implementation of the study protocol, all investigators, study coordinators, and collaborating care providers will receive standardized training before participant enrollment. Periodic monitoring visits, supervisory meetings, and protocol audits will be conducted throughout the trial to identify and address protocol deviations.

Participant adherence will be evaluated using multiple complementary measures. Caregivers will receive verbal and written instructions regarding medication administration and continuation of conventional therapy. Compliance with the investigational products will be assessed through caregiver-maintained medication diaries, reconciliation of medications dispensed and returned, and inspection of returned medication containers at each follow-up visit.

Attendance at scheduled conventional therapy sessions and study visits will be documented throughout the intervention period. Caregivers will also be interviewed regarding adherence to home-based behavioral recommendations. Participants demonstrating suboptimal adherence will receive additional counseling and reinforcement regarding the importance of treatment compliance.

Telephone reminders between scheduled visits will be used to encourage adherence, identify missed doses or therapy sessions, address caregiver concerns, and facilitate timely follow-up. An interdisciplinary team comprising Ayurvedic physicians, pediatricians, psychologists, and study coordinators will review participants’ progress at each visit and implement appropriate measures to optimize protocol adherence and participant retention.

### Concomitant Care

Participants may continue to receive routine medical treatment for conditions unrelated to ASD during the study period. However, the initiation of additional pharmacological interventions specifically intended to modify the core symptoms of ASD will not be permitted after enrollment.

If clinically indicated, investigators may prescribe supportive treatment that is considered necessary for participant safety and routine medical care. All concomitant medications and therapies, including the indication, dosage, frequency, route of administration, and duration of treatment, will be recorded in the case report form (CRF) and reviewed throughout the study to identify potential protocol deviations or confounding factors.

### Discontinuation and Modification Criteria for Allocated Interventions

Study treatment may be discontinued in the event of a serious adverse event, clinically significant intolerance, participant withdrawal of consent, or persistent nonadherence to the study protocol. Temporary dose interruption or modification may be considered for minor adverse events (AEs) at the investigator’s discretion, provided participant safety is maintained. All treatment modifications, discontinuations, and their underlying reasons will be documented in the CRFs.

### Participant Withdrawal

Participation in the study is entirely voluntary. Parents or legal guardians may withdraw their child from the study at any time without affecting future medical care or access to conventional therapy. Participants who withdraw from the study will discontinue the investigational intervention, and the reason for withdrawal, when available, will be documented.

### Sample Size

The sample size for this study was calculated based on the primary outcome measure, the ATEC score. Using G*Power software (Heinrich Heine University Düsseldorf), the required sample size was estimated with ATEC as the primary end point. A previous study on children with autism reported mean ATEC scores of 78.70 (SD 21.49) in the control group and 60.40 (SD 23.57) in the treatment group [13]. Based on these values, a sample size of 40 participants per group (total N=80) was required to detect a moderate-to-large effect size (Cohen *d* ≥0.81), with a 2-tailed α of .05% and 90% power, using an independent *t* test. This includes an adjustment for an anticipated 20% dropout rate.

### Randomization and Allocation Concealment

#### Sequence Generation

Eligible participants whose parent or legal guardian provides written informed consent and who provide age-appropriate assent will be randomly assigned in a 1:1 ratio to either the intervention group (conventional therapy plus *Saraswata Ghrita* and *Ayush Manas*) or the control group (conventional therapy alone).

#### Allocation Concealment

The random allocation sequence will be generated using computer-generated random numbers by an independent statistician who is not involved in participant recruitment, clinical assessment, or outcome evaluation. Sequentially numbered, opaque, sealed envelopes containing the allocation assignments will be prepared before participant enrollment to ensure allocation concealment.

### Implementation

Following confirmation of eligibility and completion of baseline assessments, the study coordinator will open the next envelope in sequence to assign the participant to the respective study group.

### Blinding

The study is open-label. Due to the nature of the intervention, participant and investigator blinding is not feasible. However, outcome assessments will be performed using standardized, validated instruments administered according to predefined procedures, and statistical analyses will be conducted using coded datasets to minimize assessment and analytical bias.

### Outcomes

Outcome measures will be collected at baseline (T0) and subsequently during the follow-up (T1-T6) visits at 30-day intervals. Periodic evaluation of clinical changes and facilitation of safety monitoring throughout the study will be possible through these scheduled assessments. The primary end point of the study is the change in the ATEC. The secondary end points include changes in the ISAA, WISC-IV, VABS (Daily Living Skills domain), and QoLA for parents. These selected outcome measures were chosen based on published evidence supporting their widespread use in ASD research and clinical practice. Collectively, these instruments provide a comprehensive, multidimensional assessment of cognitive and adaptive functioning in children with ASD, along with a measure of the impact on parents’ quality of life (Table 2). However, a separate psychometric validation of these instruments within this study population was not performed. Additionally, inflammatory biomarkers (hs-CRP, IL-6, and TNF-α) will be evaluated as exploratory mechanistic outcomes to investigate potential modulation of immune and inflammatory pathways implicated in ASD following adjunctive Ayurvedic therapy. Vitamin D, vitamin B12, and serum iron will be measured as part of the baseline clinical and biochemical characterization of the study population and for exploratory analyses. The trial is not designed to evaluate correction of nutritional deficiencies as a therapeutic mechanism.

**Table 2.** Primary and secondary end points with time points for each outcome.

End point	Time point
Primary outcome: efficacy	
Autism Treatment Evaluation Checklist (ATEC)	On the day of screening and 180th day
Secondary outcome: efficacy	
Indian Scale of Assessment of Autism (ISAA)	On the day of screening and 180th day
Wechsler Intelligence Scales for Children (WISC)	Baseline (day 0) and 180th day
Vineland Adaptive Behavior Scales (Daily Living Skill subscale)	Baseline (day 0) and 180th day
Quality of Life in Autism Questionnaire (QoLA) parent version	Baseline (day 0) and 180th day

### Measurements

#### ATEC

ATEC [23-25] is a diagnostic assessment tool developed by Rimland and Edelson [26], with a 77-item scale, and it was originally designed to evaluate the effectiveness of autism treatments. It is a parent-administered tool that takes around 10 to 15 minutes to complete. Multiple research studies have shown that ATEC is a reliable and valid instrument for assessing symptoms and improvements of autism symptoms in children. It has been found to be successful in measuring intervention effects and tracking behavioral development over periods of time. However, the cross-cultural validity of the ATEC has yielded mixed results.

ATEC consists of four subscales: (1) speech/language/communication, (2) sociability, (3) sensory/cognitive awareness, and (4) health/physical/behavior. The total score on this scale ranges from 0 to 179, with lower scores indicating less severe symptoms of ASD and higher scores indicating more severe symptoms of ASD. The scale provides information on any changes that may occur in the subscales over time. In (1) and (3), an answer of “not true/descriptive” receives 2 points, an answer of “somewhat true/descriptive” receives 1 point, and an answer of “very true/descriptive” receives 0 points, while in (3) an answer of “not true/descriptive” receives 0 points, an answer of “somewhat true/descriptive” receives 1 point, and an answer of “very true/descriptive” receives 2 points. In (4), an answer of “not a problem” receives 0 points, an answer of “minor problem” receives 1 point, an answer of “moderate problem” receives 2 points, and an answer of “serious problem” receives 3 points.

#### WISC-IV (India Edition)

WISC-IV [27] is an intelligence test administered to children between the ages of 6 and 16. The original WISC was developed by David Wechsler. WISC-IV provides 4 primary index scores, namely Verbal Comprehension Index (VCI), Perceptual Reasoning Index (PRI), Working Memory Index, and Processing Speed Index. These indices help recognize a child’s discrete cognitive abilities in different domains. These scores can provide the overall level of intelligence or Full-Scale IQ (FSIQ). It consists of 15 subtests, of which 10 are considered as core subtests, while the remaining 5 are supplementary. This intelligence test takes around 65 to 80 minutes to administer.

#### VABS-III

VABS-III [28] is an informant-reported scale designed to comprehensively record different aspects of adaptive functioning. It consists of domains and subdomains. VABS-III is designed to assess individuals from birth to 90 years of age. For this study, the VABS-III Daily Living Skills scale is used, which has a total of 143 items, consisting of subdomains, namely personal, domestic, and community. Each item is rated on a 3-point Likert scale (0=never, 1=sometimes, and 2=usually). The higher the rating, the better the performance/adaptive functioning, while the lower the rating, the poorer the performance/adaptive functioning.

#### ISAA

ISAA [29] is a tool for persons with autism that uses observation and clinical evaluation of the behavior, along with information given by parents or caretakers, to diagnose autism. ISAA has 6 domains, namely, social relationship and reciprocity, emotional responsiveness, speech-language and communication, behavior patterns, sensory aspects, and cognitive component. This tool consists of 40 items rated on a 5-point scale ranging from 1 (never) to 5 (always).

#### QoLA-Parent Version Questionnaire

The QoLA-parent version questionnaire [30] is used to assess the parents’ or caregivers’ well-being and overall quality of life. It includes health and non–health-related domains of functioning. This scale assesses the difficulties faced by parents of individuals with ASD. It has 2 parts. Part A has 28 items that assess their perception of their quality of life. Part A scores can range from 28 to 140, with higher scores indicating greater perceived quality of life. Part B was designed to assess parents’ perception of how challenging their child’s autism-specific difficulties are for them. Scores can range from 20 to 100, with higher scores indicating fewer perceived difficulties for the parents.

#### Indian Autism Screening Questionnaire (IASQ)

IASQ [31] is a 10-item questionnaire, which is used to assess potential autism-related symptoms in individuals. A “yes” response to any of the item is considered a positive screen and should prompt further detailed evaluation for autism. This scale is administered to parents or primary caregivers of children with autism.

The study employs standardized instruments to comprehensively assess behavioral, cognitive, adaptive, and quality-of-life outcomes in children with ASD. The ISAA is a validated instrument developed for the Indian population. The WISC-IV, VABS-III, and QoLA questionnaire are internationally established instruments with documented reliability and validity in ASD research. The ATEC has demonstrated acceptable psychometric properties, including reliability and responsiveness in previous studies, and has been widely used in longitudinal ASD research, including studies involving Indian participants [23,24,26]. The selection of these instruments was based on their established use in ASD research and their ability to comprehensively evaluate the multidimensional outcomes relevant to the objectives of the present trial.

### Benefits

The study participants may derive potential benefits from the integrative approach in terms of improvements in intellectual and adaptive functioning.

### Harms

The safety of the study intervention is assessed by documenting any AEs at each scheduled visit. All AEs occurring during the study period are recorded and monitored in accordance with GCP guidelines. Safety evaluations also include hematological and biochemical investigations, as specified.

### Participant Timeline

Participants will attend 7 scheduled visits over 6 months: one baseline visit for screening, enrollment, and initial assessments and 6 follow-up visits (days 30, 60, 90, 120, 150, and 180) for intervention, outcome assessments, and laboratory investigations. A 7-day window period will be allowed for each visit, and parents/guardians will report any AEs immediately (Figure 1).

**Figure 1.**
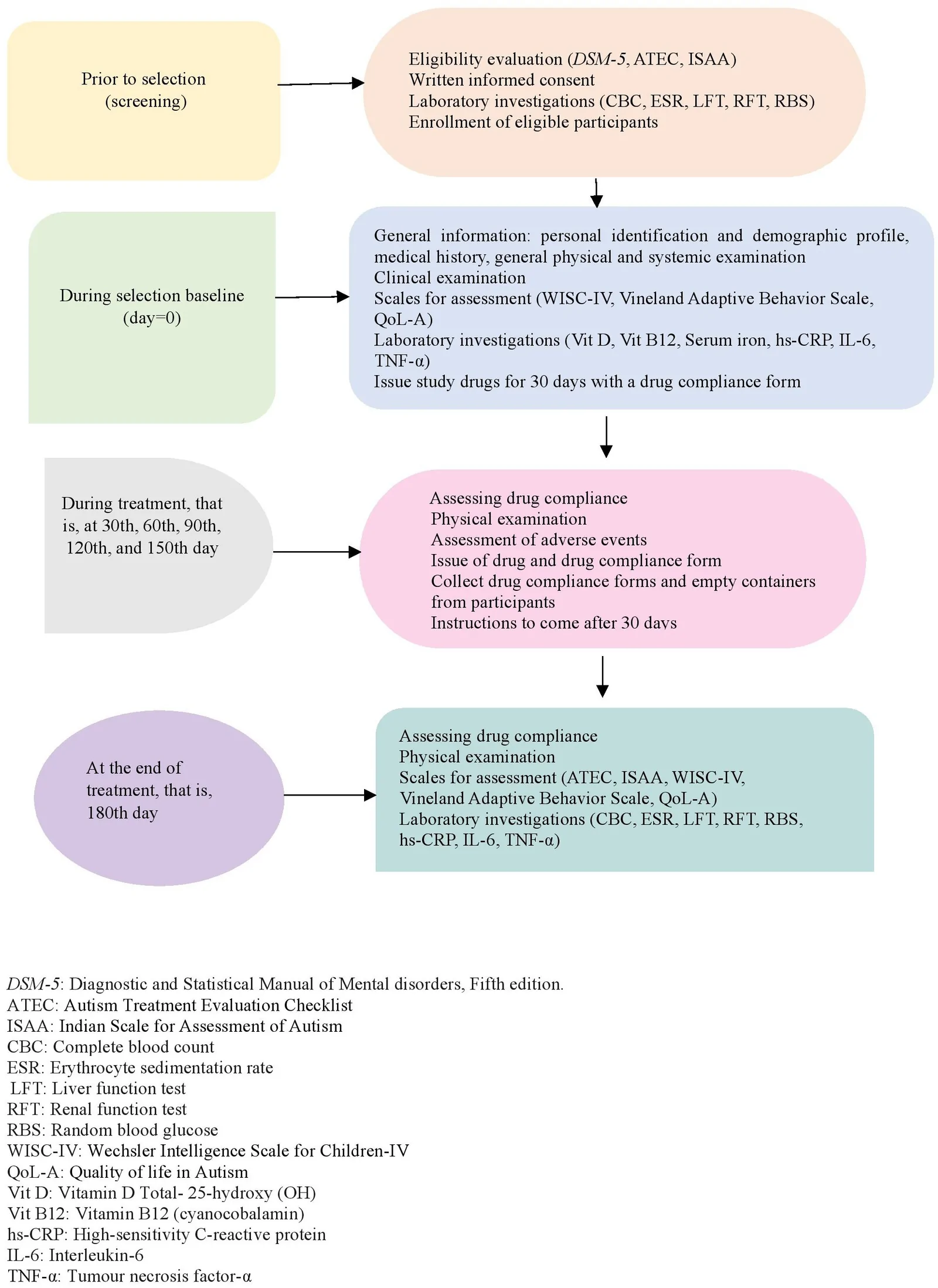
Timeline of the study. ATEC: Autism Treatment Evaluation Checklist; CBC: complete blood count; *DSM-5*: *Diagnostic and Statistical Manual of Mental Disorders, Fifth Edition*; ESR: erythrocyte sedimentation rate; hs-CRP: high-sensitivity C-reactive protein; IL-6: interleukin-6; ISAA: Indian Scale for Assessment of Autism; LFT: liver function test; QoLA: Quality of Life in Autism; RBS: random blood glucose; RFT: renal function test; TNF-α: tumor necrosis factor-α; vit B12: vitamin B12 (cyanocobalamin); vit D: vitamin D, total 25-hydroxy (OH); WISC: Wechsler Intelligence Scale for Children.

### Data Collection and Management

Baseline demographic, clinical, laboratory, and outcome assessment data will be recorded in standardized CRFs by trained study personnel. Data collected during follow-up visits, including treatment adherence, concomitant medications, AEs, and outcome measures, will be entered promptly into the study database.

All study records will be reviewed for completeness and accuracy before data entry. Double data entry, periodic data verification, and quality-control procedures will be implemented to minimize transcription errors and ensure data integrity. Data queries and discrepancies will be resolved through source document verification.

Participant confidentiality will be maintained by assigning a unique study identification number to each participant. Personal identifiers will be stored separately from study data, and access to identifiable information will be restricted to authorized study personnel. Electronic data will be maintained in password-protected computers with regular data backups, while hard-copy records will be stored in locked cabinets within secured research facilities.

Study documents will be retained in accordance with institutional policies and applicable regulatory requirements upon completion of the trial.

### Statistical Analysis

#### Statistical Methods

The statistical analysis will be conducted after data collection, with verification of its accuracy and limitations. All randomized participants will be analyzed according to group allocation. Normality of the data will be checked using the Shapiro-Wilk test. Categorical variables will be presented as numbers and percentages, while continuous variables will be presented as mean (SD) for normally distributed data and as median with IQR (Q1-Q3) for nonnormally distributed data. Preintervention and postintervention comparisons of categorical variables will be analyzed using the chi-square test. Continuous variables, such as scores and laboratory parameters, will be analyzed using the paired or unpaired 2-tailed *t* test for normally distributed data, or the Wilcoxon signed-rank test for nonnormally distributed data. For assessment parameters measured across multiple follow-up points, repeated measures ANOVA (repeated measures ANOVA) will be used for normally distributed data, while the Friedman test will be used for nonnormally distributed or categorical data. All statistical tests will be conducted at a 5% level of significance (*P*<.05). Analyses will be carried out using STATA software (version 18.0). Missing data will not be imputed.

#### Safety Monitoring

Safety assessments will include clinical evaluation, laboratory investigations, and documentation of AEs throughout the study period. All AEs will be recorded at each follow-up visit and classified according to severity, seriousness, duration, outcome, and relationship to the study intervention.

Serious AEs will be reported promptly to the Institutional Ethics Committee (IEC) and the study sponsor in accordance with applicable regulatory requirements. Participants who develop clinically significant AEs will receive appropriate medical management, and continuation of the study intervention will be determined by the principal investigator based on clinical judgment and participant safety.

### Ethical Considerations

#### Research Ethics Approval

The study received approval from the IEC of the CARI in Bengaluru prior to participant recruitment. The trial has also been registered with the Clinical Trials Registry–India (CTRI/2025/04/085035).

#### Assent

Written informed consent will be obtained from the parent or legally authorized guardian of each participant before any study-specific procedures are undertaken. When developmentally appropriate, age-appropriate assent will also be obtained from participating children.

#### Privacy and Confidentiality

Participation is voluntary, and parents or legal guardians may withdraw their child from the study at any stage without affecting access to routine medical care or conventional therapy. Confidentiality of participant information will be maintained throughout the study, and study findings will be disseminated in aggregate form without revealing individual participant identities.

#### Protocol Amendments

Any deviations from the study protocol will be implemented only after obtaining prior approval from the IEC and the sponsoring body, CCRAS. The nature and justification for each deviation will be documented in the CRF, source documents, and monitoring visit reports. Appropriate corrective and/or preventive actions will be taken and recorded, which may include retraining of the investigator and study staff as needed. Any significant modifications to the study will be incorporated through formally approved protocol amendments.

The study will be conducted in accordance with the ethical principles of the Declaration of Helsinki, the International Council for Harmonisation-GCP (ICH-GCP) guidelines, and the applicable ethical and regulatory requirements governing clinical research in India.

#### Dissemination

The study findings will be published by the sponsor in a scientific peer-reviewed journal. A lay summary of the results will be provided to study participants.

#### Ancillary and Posttrial Care

Clinical trial insurance coverage will be provided to participants in the event of any unanticipated risks or critical medical conditions arising during the study. All participants will receive appropriate posttrial assistance or, if required, be referred to the study center for further evaluation and management.

## Results

As of July 17, 2026, a total of 20 participants had been enrolled in the study and completed baseline assessments. The mean age of the enrolled participants was 9.7 (SD 2.7) years, with 16 (80%) being male participants. The mean baseline ATEC score was 57.6 (SD 20.0), while the mean ISAA score was 93.5 (SD 16.0), indicating enrollment of participants with mild-to-moderate ASD in accordance with the study eligibility criteria. Owing to the heterogeneous cognitive and behavioral profiles and individual variability among children with ASD, domain-specific scores are being derived and analyzed for each participant. Consequently, the mean baseline WISC-IV FSIQ scores are being analyzed. The mean baseline score of VABS-III Daily Living Skills was 61.7 (SD 19.1), and QoLA-parent version score part A and part B were 101.0 (SD 19.9) and 61.3 (SD 14.5), respectively (Table 3). These data are presented solely to describe the characteristics of the study cohort; no interim efficacy, safety, or comparative analyses have been performed, as participant recruitment and follow-up have not been completed (Figure 2).

**Table 3.** Baseline characteristics of participants enrolled as of July 17, 2026 (N=20).

Characteristics	Values (N=20)
Demographic characteristics	
Age (y), mean (SD)	9.7 (2.7)
Male sex, n (%)	16 (80)
Female sex, n (%)	4 (20)
Weight (kg), mean (SD)	33.5 (11.5)
Height (cm), mean (SD)	1.38 (0.16)
BMI (kg/m²), mean (SD)	17.2 (3.4)
Autism-related characteristics	
Age at diagnosis (y), mean (SD)	3.0 (2.2)
Duration since diagnosis (y), mean (SD)	7.6 (3.0)
Functional and cognitive assessments	
ISAA^a^ score, mean (SD)	93.5 (16.0)
Mild ASD^b^ (ISAA 70‐106), n (%)	14 (70)
Moderate ASD (ISAA 107‐153), n (%)	6 (30)
ATEC^c^ total score, mean (SD)	57.6 (20.0)
VABS-III^d^ Daily Living Skills score, mean (SD)	61.7 (19.1)
QoLA^e^ (parent version), mean (SD)	
Part A	101.0 (19.9)
Part B	61.3 (14.5)

a ISAA: Indian Scale for Assessment of Autism.

b ASD: Autism Spectrum Disorder.

c ATEC: Autism Treatment Evaluation.

d VABS: Vineland Adaptive Behavior Scales.

e QoLA: Quality of Life in Autism.

**Figure 2.**
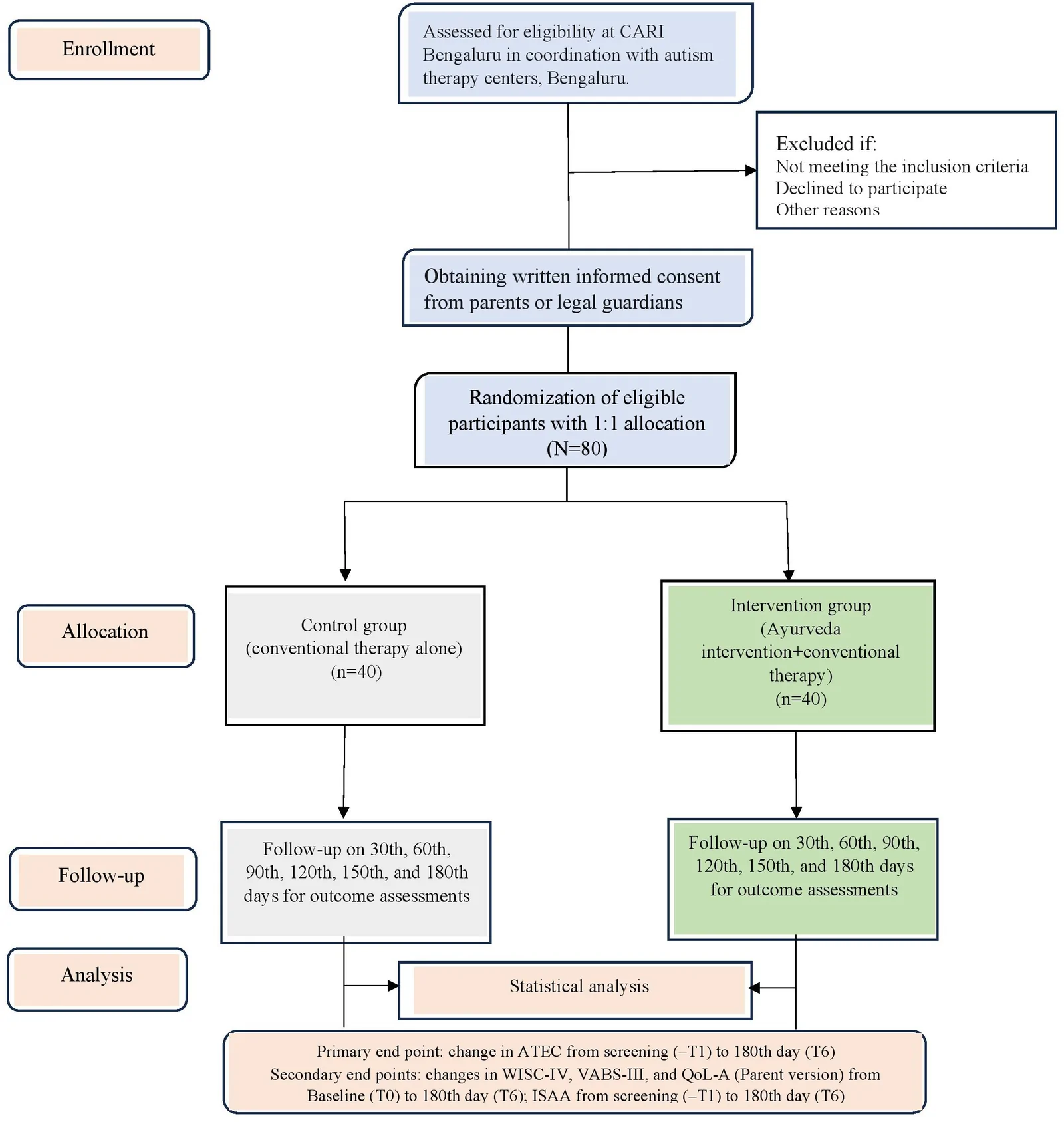
CONSORT (Consolidated Standards of Reporting Trials) flow diagram. This CONSORT flowchart illustrates the planned participant recruitment for this study protocol. ATEC: Autism Treatment Evaluation Checklist; CARI: Central Ayurveda Research Institute; ISAA: Indian Scale for Assessment of Autism; QoLA: Quality of Life in Autism; VABS: Vineland Adaptive Behavior Scales; WISC: Wechsler Intelligence Scale for Children.

## Discussion

### Principal Considerations

ASD is a complex neurodevelopmental condition that affects multiple domains of functioning, including social communication, behavior, cognition, and adaptive skills. Although multidisciplinary conventional interventions remain the cornerstone of management, many children continue to experience persistent functional limitations despite prolonged therapy. Consequently, there is increasing interest in adjunctive interventions that target multiple biological and behavioral pathways while complementing established therapeutic approaches.

### Comparison With Prior Work

This protocol describes a randomized, open-label, active-controlled clinical trial designed to evaluate the efficacy of an integrative treatment approach combining *Saraswata Ghrita* and *Ayush Manas* with conventional therapy for children with ASD. Unlike previous studies that have primarily focused on behavioral outcomes, the present trial adopts a multidimensional evaluation strategy incorporating behavioral symptoms, intellectual functioning, adaptive behavior, caregiver quality of life, and exploratory inflammatory biomarkers. This comprehensive assessment is intended to provide a broader understanding of the potential clinical and biological effects of adjunctive Ayurvedic therapy [32].

The inclusion of inflammatory biomarkers, including hs-CRP, IL-6, and TNF-α, is based on growing evidence suggesting that immune dysregulation and neuroinflammation may contribute to ASD pathophysiology in a subset of individuals. Although these biomarkers are not intended as diagnostic or surrogate end points, their assessment may provide preliminary insights into biological mechanisms associated with treatment response and help inform future mechanistic studies [33-36].

The study also incorporates validated instruments to assess multiple dimensions of ASD, including autism severity, cognitive performance, adaptive behavior, and caregiver quality of life. This multidimensional outcome framework is expected to provide a more comprehensive evaluation of intervention effects than reliance on behavioral measures alone.

### Strengths

This study has several methodological strengths. It is among the few RCTs evaluating Ayurveda as an adjunct to conventional therapy for children with ASD. The protocol follows SPIRIT recommendations and incorporates standardized eligibility criteria, predefined outcome measures, and structured follow-up assessments to enhance methodological rigor.

Another strength is the integration of validated behavioral, cognitive, adaptive, caregiver-reported, laboratory, and safety outcomes. Simultaneous evaluation of these complementary domains enables a comprehensive assessment of treatment effects while maintaining participant safety throughout the study.

Treatment adherence is supported through standardized caregiver education, medication diaries, drug accountability procedures, scheduled follow-up visits, and regular communication with participating families. These measures are expected to improve intervention fidelity and data quality.

### Limitations

Owing to the nature of the interventions, participant and investigator blinding was not feasible. This may have introduced the potential for performance and observer bias, which is acknowledged as a limitation of the study. However, standardized assessment procedures and predefined statistical analyses have been incorporated to minimize bias.

The study is being conducted at a single coordinating center with collaboration from selected autism therapy centers, which may limit the generalizability of the findings to broader populations. In addition, variability in the intensity and frequency of conventional therapies across participants may influence treatment outcomes despite careful documentation of concomitant interventions.

The exploratory biomarker analysis is intended to generate hypotheses regarding potential biological mechanisms rather than establish causal relationships. Consequently, any observed biomarker changes should be interpreted cautiously and confirmed in future adequately powered mechanistic studies.

The present study did not include a separate psychometric validation of the outcome measures, particularly the ATEC, in the study population prior to trial implementation. Although all assessment tools used in the study have established validity and reliability in previous research, their psychometric properties were not specifically evaluated in this cohort. Future studies should consider formal psychometric validation, including assessment of internal consistency, test-retest reliability, and construct validity, to further strengthen the interpretation and generalizability of the findings.

### Implications for Future Research

The findings of this trial will contribute to the limited evidence base regarding integrative management of ASD. If the intervention demonstrates acceptable safety and promising clinical benefits, the results will support the design of larger multicenter RCTs with longer follow-up periods and broader participant populations.

Future studies may also incorporate advanced neuroimaging, genomic, metabolomic, and microbiome analyses to further elucidate the biological mechanisms underlying treatment response and identify predictors of therapeutic benefit. Such investigations could facilitate the development of more individualized, integrative treatment strategies for children with ASD.

### Conclusion

The study makes a significant step toward the evidence-based integration of Ayurveda with conventional therapy in managing ASD. By integrating validated assessments of behavioral symptoms, intellectual functioning, adaptive behavior, caregiver quality of life, and exploratory inflammatory biomarkers, the study adopts a comprehensive approach to evaluating both the clinical and potential biological effects of the intervention. The findings of the trial are expected to contribute to the limited high-quality evidence regarding the role of Ayurveda as an adjunct to conventional ASD management. If shown to be safe and beneficial, this integrative approach may support the development of evidence-based multidisciplinary treatment strategies and inform future multicenter clinical trials investigating Ayurvedic interventions for neurodevelopmental disorders.

## Supplementary material

10.2196/99910 Checklist 1 SPIRIT 2025 checklist.

10.2196/99910 Peer Review Report 1 Peer review report from the Central Council for Research in Ayurvedic Sciences.

## Abbreviations

AE: adverse event
API: Ayurvedic Pharmacopeia of India
ASD: Autism Spectrum Disorder
ATEC: Autism Treatment Evaluation Checklist
CARI: Central Ayurveda Research Institute
CCRAS: Central Council for Research in Ayurvedic Sciences
CRF: case report form
CTRI: Clinical Trial Registry of India
*DSM-5*: *Diagnostic and Statistical Manual of Mental Disorders, Fifth Edition*
FSIQ: Full-Scale IQ
GCP: Good Clinical Practice
GMP: Good Manufacturing Practice
hs-CRP: high-sensitivity C-reactive protein
IASQ: Indian Autism Screening Questionnaire
*ICD-11*: *International Classification of Diseases, 11th Revision*
ICH: International Council for Harmonisation
IEC: Institutional Ethics Committee
IEP: Individualized Education Program
IL-6: interleukin-6
ISAA: Indian Scale for Assessment of Autism
PRI: Perceptual Reasoning Index
QoLA: Quality of Life in Autism
RCT: randomized controlled trial
SPIRIT: Standard Protocol Items: Recommendations for Interventional Trials
TNF-α: tumor necrosis factor-alpha
VABS: Vineland Adaptive Behavior Scales
VCI: Verbal Comprehension Index
WISC: Wechsler Intelligence Scale for Children
