# Supplementary material for: Multidimensional Evaluation of the Efficacy of a Comprehensive Ayurveda Approach as an Add-On to Conventional Therapy in Children With Autism Spectrum Disorder: Protocol for a Randomized, Open-Label, Parallel-Group, Active-Controlled Clinical Trial
Source: JMIR Res Protoc. 2026 Oct 9;15:e99910. doi: 10.2196/99910 (PMC13652383; doi:10.2196/99910)
Supplement: Supplementary file 2 [file resprot-v15-e99910-s002.pdf]

Peer review report

The Central Council for Research in Ayurvedic Sciences (CCRAS), an autonomous organization under the Ministry of AYUSH, Government of India, is the apex body in India dedicated to undertaking, coordinating, formulating, developing, and promoting scientific research in Ayurvedic Sciences. Any research project sanctioned by CCRAS undergoes a rigorous scientific and administrative evaluation process. The project proposal is initially reviewed at the Internal Scrutiny Committee (ISC) meeting, followed by evaluation at the Project Evaluation Monitoring Committee (PEMC) meeting. During these stages, the proposals are critically examined for their scientific merit, methodology, feasibility, ethical aspects, and relevance to public health. The investigators are required to revise and refine the proposals based on the committees' observations and recommendations before final approval and sanction of the project. The submitted project has been presented in both ISC meeting and PEMC meeting and peer review of the same was conducted. The documents are as follows

26 DEC 2023

केन्द्रीय आयुर्वेदीय विज्ञान अनुसंधान परिषद्
(आयुष मंत्रालय, भारत सरकार की स्वायत्त निकाय)
जवाहर लाल नेहरू भारतीय चिकित्सा एवं होम्योपैथी अनुसंधान भवन,
61–65, इंस्टीट्यूशनल एरिया, डी ब्लॉक के सामने, जनकपुरी, नई दिल्ली–110058
Central Council for Research in Ayurvedic Sciences
(An Autonomus Body of Ministry of AYUSH, Govt. of India)
Jawahar Lal Nehru Bhartiya Chikitsa Evam Homoepathy Anusandhan Bhawan,
61-65, Institutional Area, Opp. 'D' Block Janak Puri, New Delhi-110058
Website: ccras.nic.in, E-mail: dg-ccras@nic.in,
EPBX.: 011-28520748, 28525950, 28522010, 28521201, 28524810, 28525095, 28520945

15 YEARS OF CELEBRATING THE MAHATMA

F.No.HQ-ADMN018/33/2022-ADMN/6519 **Date:**

Sub: Minutes of the 3 Internal Scrutiny Committee (ISC) meeting viz. 16th, 17th & 18th held on 22.11.2023, 01.12.2023 & 6.12.2023 in hybrid mode at committee room, CCRAS Hqrs. New Delhi- reg.

Sir/Madam,

Please find enclosed herewith the Minutes of the 3 Internal Scrutiny Committee (ISC) meeting viz. 16th, 17th & 18th held on 22.11.2023, 01.12.2023 & 6.12.2023 in hybrid mode at committee room, CCRAS Hqrs., New Delhi for information and necessary action.

Yours Faithfully,

Encl. as above

26/12/2023

(Renu Rajan)
Administrative Officer (Project)
For Director General
#ApprovedBy

To,

All concerned officer as per list (Annexure-I)

Copy to:

1. All Concerned Programme Officers/ Nodal Officers of CCRAS Hqrs. For information and necessary action.
2. Sr. PS to DG CCRAS, New Delhi.

26/12/2023

(For Director General)

ANNEXURE-I

List of officers/officials who attend the 16th ISC meeting of (Clinical Research) to held at committee room, CCRAS Hqrs. On 22nd Nov. 2023 at 10:00 AM

1. Dr. N. Srikanth, Deputy Director General, CCRAS
2. Dr. Atul Juneja, Former Scientist 'F', ICMR-NIMS
3. Shri. Deepak Kocchar, Deputy Director Admin, CCRAS
4. Dr. Sanjaya Kumar YR, Assistant Director(Pharmacology)
5. Dr. B.C.S Rao, Assistant Director(Ay.), CCRAS
6. Dr. Renu Makhija, Assistant director (Path.), CCRAS
7. Dr. Pratap Makhija, Assistant Director(Ay.), CCRAS
8. Dr. T. Maheshwar, Assistant Director(Ay.), CCRAS
9. Dr. G. Nartunai, Assistant Director(P'cognosy), CCRAS
10. Dr. Deepa Makhija, Assistant Director(Ay.), CCRAS
11. Dr. Sunita, Research Officer(Ay.), CCRAS
12. Dr. Renu Singh, Research Officer(Ay.), CCRAS
13. Dr. A.K. Jain, Research Officer(Ay.), CCRAS
14. Dr. Shruti Khanduri, Research Officer(Ay.), CCRAS
15. Dr. Rakesh Rana, Statistical Officer, CCRAS
16. Dr. Vinod Lavaniya, Research Officer(Ay.), CCRAS
17. Dr. B.S Sharma, Research Officer(Ay.), CCRAS
18. Dr. Arunabh Tripathi, Statistical Officer, CCRAS
19. Dr. Azeem Ahmed, Research Officer(Ay.), CCRAS
20. Dr. Pallavi Mundada, Research Officer(Ay.), CCRAS
21. Dr. Sophia, Research Officer(Ay.), CCRAS
22. Dr. Amit K. Rai, Research Officer(Ay.), CCRAS
23. Dr. Ashwathy K. Research Officer(Ay.), CCRAS, joined online
24. Dr. Lalita Sharma, Research Officer(Ay.), CCRAS joined online
25. Dr. Prashant Shinde, Research Officer(Ay.), CCRAS
26. Dr. Kalpana Kachare, Research Officer(Ay.), CCRAS joined online

CENTRAL COUNCIL FOR RESEARCH IN AYURVEDIC SCIENCES

Minutes of 17th meeting of the Internal Scrutiny Committee held on 01.12.2023th at 10.00 AM

The 17th meeting of the Internal Scrutiny Committee was held 01.12.2023 at 10.00 AM under the Chairmanship of Dr N. Srikanth, DDG, CCRAS in hybrid mode at CCRAS Headquarters to discuss 15 Research project proposals (14 Clinical Research & 01 Pharmacology) submitted from the CCRAS Institutes. The list of officers who attended the meeting excluding the investigators who presented the proposals is at **Annexure-II**. Some general comments were suggested during the meeting as follows:

- Institutes shall ensure that they submit the concept proposals with all relevant fields such as objectives, outcomes, sample size (calculated in consultation with biostatistician) along with justification, methodology and budget.
- The information regarding trial interventions shall be complete with API reference, classical references, availability of standards, and pharmacy identified for procurement
- Cross sectional studies should also have specific details regarding the population trends, sampling frame, sample size, detailed methodology, statistical methods and draft questionnaire.
- The standard of care (SoC) interventions shall be finalized by concerned specialists.

Following are the comments of the committee after thorough discussion on the proposals –

Sl. No.	Project title	PI & Institute	Comments of the ISC
		**CLINICAL RESEARCH**	
1.	A Double-blind, placebo-controlled, Randomised prospective study to evaluate the efficacy of Ayurveda formulation and Nutritional supplement in children with Autism Spectrum Disorder	**Dr Vidyashree Anchan** MBBS, MD (Pathology), Research Officer (Pathology)	**1.** The study could undergo a modification, incorporating two groups: one receiving conventional care and the other adopting the Ayurveda comprehensive approach.<br>2. The Ayurvedic approach tailored for Autism Spectrum Disorder is proposed to be finalized in consultation with CCRAS headquarters.<br>3. In alignment with the revised approach, the objectives and outcome measures of the study may be appropriately adjusted<br>4. The sample size may also be re-calculated in consultation with the bio-statistics team at CCRAS Hqrs, ensuring that suitable power is maintained.<br>5. Validation of Autism Evaluation Checklist (ATEC) for use in Indian population is proposed as an objective in the proposed study. However, it should be done as a Psychometric Validation Study, which is not under the purview of the current study

Sl. No.	Project title	PI & Institute	Comments of the ISC
			and the same may be proposed separately to assess the reliability, validity, and other psychometric properties of the tool, to ensure its appropriateness and effectiveness in the specific population of interest.<br>6. The feasibility of enrolling children based on the specified selection criteria is pivotal for the successful execution of the study. Therefore, it is imperative to secure written agreement for the participation/referral of eligible participants from potential recruitment sites, which are envisioned as therapy centers for Autism Spectrum Disorders. This step should be duly incorporated and outlined in the revised protocol.<br>7. To ensure that participants, once recruited are consistently receiving conventional care, including behavioral therapy, occupational therapy, speech therapy, etc., from the designated referral sites, a dedicated team comprising social workers, psychologists, therapists, and other relevant professionals may need to be engaged and the budgetary aspects, may rationally be incorporated into the study proposal. Recognizing the significance of therapy compliance, monitoring and compliance to therapeutic intervention, shall be inbuilt in the protocol.<br>**Overall Impression: The proposal may be revised and resubmitted within 07 days addressing all the aforementioned comments.**

CENTRAL COUNCIL FOR RESEARCH IN AYURVEDIC SCIENCES

Minutes of the 10th PEMC meeting held on 27th March, 2024 at 10.00AM at CCRAS Headquarters in hybrid mode

The 10th meeting of the Project Evaluation Monitoring Committee (PEMC) was held on 27th March, 2024 in hybrid mode at 10.00 AM under the chairmanship of Prof.Vaidya Rabinarayan Acharya, Director General CCRAS. The details of committee members and CCRAS officers who attended the meeting is annexed at **Annexure** I.

At the outset, Dr.N.Srikanth, DDG, CCRAS welcomed all committee memmbers and briefed about the proposed 17 agenda items., viz.,13 clinical research, 02 public health and 02 fundamental research projects. Following which presentations were made by respective principal investigators. The recommendations of the PEMC are as follows:

General Recommendations:

1. The budget for the proposals should undergo rationalization before submission to PEMC. The inclusion of contractual staff in the studies may be proposed only if adequately justified. In the case of a clinical trial, rationalizing the budget shall involve a thorough assessment of expenses related to research, such as procurement of interventions, participant recruitment, site management, data collection and analysis,insurance, and any other pertinent factors to ensure tcost-effectiveness and optimal resource allocation.
2. The Principal Investigator (PI) is required to emphasize the indications for trial interventions and provide a comprehensive list of potential adverse effects or contraindications associated with them in the protocol.
3. For clinical studies, the Case Report Form (CRF) must be included at the time of submission for consideration by PEMC. For observational studies, a draft questionnaire must also be submitted.
4. The Principal Investigator (PI) must ensure a thorough understanding of the statistical assumptions used for sample size calculation so that they can confidently present the same to the PEMC.
5. The revised proposals shall be submitted to CCRAS hqrs within 15 days of issue of the minutes.

The detailed list of projects is at **Annexure – II**.

At the end of meeting, Chairman PEMC - DG, CCRAS expressed sincere thanks to the members for extending their support, encouragement and offering valuable suggestions.

The meeting ended with vote of Thanks

Annexure-I

List of Committee Members and CCRAS Officers who attended the meeting :

1. Prof. Rabinarayan Acharya, Director General, CCRAS, New Delhi
2. Dr. S. K. Benara, Former Scientist F, ICMR-NIMS, New Delhi
3. Prof. Prasanna Narasimha Rao, Principal, SDM College of Ayurveda & Hospital, Hassan, Karnataka
4. Prof. A.C. Kar, Professor, Department of Vikriti Vigyan, IMS, BHU Varanasi
5. Dr. Arun Gupta, Professor Panchakarma, CBPACS, Delhi
6. Dr. Atul Juneja, Former Scientist F, (ICMR-National Institute of Medical Statistics, Ansari Nagar, New Delhi), A2B/115B, Ekta Appartment, Paschim Vihar, New Delhi-63
7. Mr. Raj Kumar, Director (IFD), Ministry of Ayush, Ayush Bhawan, B Block, GPO Complex, INA Colony, New Delhi (Representative from Internal Finance Division of Ministry of Ayush)
8. Dr. N. Srikanth, Deputy Director General, CCRAS, New Delhi
9. Dr. BCS Rao, Assistant Director (Ay.), CCRAS Hqrs, New Delhi
10. Dr. Deepa Makhija, Assistant Director (Ay.), CCRAS Hqrs, New Delhi
11. Dr. B.S. Sharma, Research Officer (Ay.), CCRAS Hqrs, New Delhi
12. Dr. Renu Singh, Research Officer (Ay.), CCRAS Hqrs, New Delhi
13. Dr. Shruti Khanduri, Research Officer (Ay.), CCRAS Hqrs, New Delhi
14. Dr. Pallavi Mundada, Research Officer (Ay.), CCRAS Hqrs, New Delhi
15. Dr. Sophia Jameela, Research Officer (Ay.), CCRAS Hqrs, New Delhi
16. Dr. Azeem Ahmad, Research Officer (Ay.), CCRAS Hqrs, New Delhi
17. Dr. Neha Dubey, Research Officer (Ay.), CCRAS Hqrs, New Delhi
18. Dr. Anagha Ranade, Research Officer (Ay.), CCRAS Hqrs, New Delhi
19. Dr. Kalpana Kachare, Research Officer (Ay.), CCRAS Hqrs, New Delhi
20. Dr. Prashant Shinde, Research Officer (Ay.), CCRAS Hqrs, New Delhi
21. Dr. Rakesh Kumar Rana, Statistical Officer, CCRAS Hqrs, New Delhi

ANNEXURE - II

Minutes of the Projects discussed in 10th PEMC held on 27th March, 2024

Agenda No	Project Name	Institute name	PI	Comments
10.1	Management of Autism Spectrum Disorder in children through a comprehensive Ayurveda approach – A randomized control clinical trial.	CARI, Bengaluru	**Dr. Vidyashree Anchan**<br>**Mob**: 8971859797 / 080 29635035<br>**Email**: dr.anchan88@gmail.com ; vidyashree@ccras.nic.in	The title should be revised to reflect that Ayurvedic intervention is adjunct to conventional care.<br>The inclusion of an almirah in the budget does not comply with the provisions outlined in the CCRAS research policy and should be omitted.<br>The budget allocation for TA/conveyance/honoraria and publication, currently consolidated under one subhead totaling Rs. 10,00,000/-, should be appropriately segregated and rationalized based on specific requirements.<br>The expenditure for insurance in year 1 and year 2 appears disproportionate, especially considering that insurance will be paid in premiums. Adjustments should be made as per the amount finalized in consulattaion with the insurance broker.<br>To finalize the budget for trial interventions, the estimated cost of medicines should be obtained from CARI-Jhansi or IMPCL, as appropriate.<br>The allocation of Rs. 10,00,000/- under contingency seems excessive for unforeseen expenses and should be rationalized<br>Given the potential challenges with the palatability of Ghrita in children with Autism, it is recommended to investigate the feasibility of utilizing modified dosage forms.<br>The conventional therapy for Autism Spectrum Disorder (ASD) and relevant existing guidelines should be incorporated into the intervention subheading. Measures must be implemented to ensure uniform therapy administration across all therapy centers participating in the study to maintain consistency.<br>The objectives related to nutritional parameters may be omitted as there is no evidence to support a direct correlation between the proposed intervention and

Agenda No	Project Name	Institute name	PI	Comments
				nutritional factors in ASD. Accordingy budget may also eb revisited.<br>**Recommendation: The project is endorsed with the recommendation that the proposal be submitted to CCRAS Headquarters for thorough review, accompanied by proposed revisions and updated budget.**
